# Knowledge of cervical tuberculosis lymphadenitis and its treatment in pastoral communities of the Afar region, Ethiopia

**DOI:** 10.1186/1471-2458-11-157

**Published:** 2011-03-09

**Authors:** Mengistu Legesse, Gobena Ameni, Gezahegne Mamo, Girmay Medhin, Gunnar Bjune, Fekadu Abebe

**Affiliations:** 1 Aklilu Lemma Institute of Pathobiology, Addis Ababa University, Addis Ababa, Ethiopia; 2 Faculty of Veterinary Medicine, Addis Ababa University, Bishofittu, Ethiopia; 3 Department of General Practice and Community Medicine, Institute for Health and Society, University of Oslo, Oslo, Norway

## Abstract

**Background:**

Infection with *Mycobacterium bovis *(Mb) predominantly causes cervical TB lymphadenitis (TBL). Raw milk is considered the main source of Mb infection and raw milk is a major food source for Afar pastoralists. The aim of this study was to assess Afar pastoralists' knowledge concerning cervical TBL and its treatment.

**Methods:**

A community-based cross-sectional survey involving 818 interviewees was conducted in two districts of the Afar Region, Ethiopia. In addition, two focus group discussions (FGDs) were conducted in each of the study areas, one with men and the other with women.

**Results:**

Of the 818 interviewees [357 (43.6%) females and 461 (56.4%) males], 742 (90.7%) reported that they had knowledge of cervical TBL, mentioning that swelling(s) on the neck resulting in a lesion and scar are common symptoms. However, only 11 (1.5%) individuals mentioned that bacteria or germs are the causative agents of TBL. Three interviewees and a male discussant mentioned drinking raw milk as the cause of TBL. A considerable proportion (34.2%) of the interviewees and almost all the discussants suggested herbal medicine as an effective treatment. Male study participants were 1.82 times more likely to have overall knowledge of TBL than female study participants (adjusted OR, 1.82; 95% CI, 1.32 to 2.51, p < 0.001).

**Conclusion:**

The pastoral community members in the study areas had little biomedical knowledge of the cause, the source of infection and the transmission route of cervical TBL. Furthermore, most community members believed that herbal medicines are the most effective treatment for TBL. Therefore, TB control programs in the Afar Region require the incorporation of public health education introducing current biomedical knowledge of the disease. In addition, further studies are important to elucidate which medicinal plants are used by Afar pastoralists to treat TBL.

## Background

Pulmonary Tuberculosis (PTB) is a major health problem in developing countries [1], caused by exposure to *Mycobacterium tuberculosis *(Mtb). Extra-pulmonary tuberculosis (EPTB) has made a profound contribution to the burden of TB, particularly since the emergence of the human immunodeficiency virus (HIV) [2], and TB lymphadenitis (TBL) is the most common form of EPTB [2-4]. Studies indicate that infection with *Mycobacterium bovis *(Mb) predominantly causes EPTB, particularly TBL [5,6], while consumption of raw milk plays a major role in Mb infection in humans [5].

According to the WHO report [1], Ethiopia is ranked 7^th ^among 22 countries world-wide with a high burden of TB, and 3^rd ^in terms of the number of EPTB cases. TB is a major public health problem in Afar pastoralists [1,7]. Epidemiological data reveal a conservative magnitude of PTB and EPTB in the Afar region, but case records concerning TB over a seven year period (2001-2007) obtained from the Afar Regional Health Bureau indicate that approximately 28% of cases were due to EPTB; TBL is the most common form of EPTB in the region.

Studies have demonstrated that lack of knowledge concerning the cause, mode of transmission, symptoms, and appropriate treatment for PTB affect the health-seeking behaviour of patients. Furthermore, this could affect control strategies, thereby sustaining transmission of the disease within the community [8-15]. Therefore, gathering information concerning a community's knowledge of TB is useful for designing effective control strategies. As part of a large on-going study concerning TB in Afar pastoralists and their livestock, a questionnaire survey was conducted to explore what knowledge pastoral communities have concerning the cause, mode of transmission, symptoms, prevention and treatment of PTB and TBL. Data concerning PTB and TBL were analyzed separately and the findings relating to PTB has been reported elsewhere [16].

## Methods

### Study area and population

Between March and May 2009, a community-based cross-sectional survey concerning the prevalence of latent and active TB in pastoralists and their livestock was conducted in the Dubti and Amibara districts of the Afar Region, North-East Ethiopia. Dubti district is located in the Lower Awash valley, approximately 574 km North-East of Addis Ababa. The district has 18 small administrative units (kebeles), of which three are towns. Amibara district is located in the Middle Awash valley, ~260 km East of Addis Ababa. It has 18 kebeles, of which four are towns/semi-towns. The study areas and the study population have been described in detail elsewhere [16].

Prior to implementing a survey concerning the prevalence of TB, the knowledge and perception of the communities to PTB and TBL was assessed. Results of the study concerning PTB have been reported elsewhere [16]. During the survey period, no previous information concerning the level of pastoral community awareness of TBL in the study areas or the region as whole were available. Therefore, on the assumption that 50% of the participants in each of the study districts had knowledge of TBL, (95% confidence and 5% degree of accuracy) and a design effect of 1.1 due to cluster sampling, we calculated that we would need 424 participants from each of the selected districts. Eligible participants were members of selected kebeles, a husband/wife (responsible person) in the selected households, apparently healthy and willing to be interviewed. The study protocol was approved by the Ethical Clearance Committee of the Aklilu Lemma Institute of Pathobiology (ALIPB), Addis Ababa University and the Regional Committee for Medical Research Ethics of Southern Norway. The aim of the study was explained to each of the participants and verbal consent was obtained. Each participant was interviewed independently and the information collected was kept confidential. In the case of refusal to participate, a person from the next household would be interviewed.

### Selection of study participants and data collection

Prior to data collection, a list of kebeles in the selected districts was obtained from the respective District Health Offices. Owing to time and financial constrains, not all the pastoral kebeles in the districts were included in the study. Therefore, seven randomly selected pastoral kebeles from the 14 pastoral kebeles of the Amibara District and six randomly selected pastoral kebeles from the 15 in the Dubti District were included. All villages in the selected kebeles were to be included in the study, but some villages were not available during the survey owing to temporary migration to other areas. Therefore, the selected kebeles were stratified into available villages (if there was more than one village in the kebele) and a list of households from each village was prepared. On the basis of the number of households in each kebele, the pre-estimated sample size (424) for each of the study districts was proportionally distributed to the study kebeles. The required number of participants (husband or wife) was systematically selected from each kebele using these lists.

Structured and open-ended questionnaires were prepared in English [Additional file 1]. For clarity and simplicity, the questionnaires were first translated into Amharic and then into the local language (Afar). The questionnaires were assessed for clarity and cultural acceptability in the study districts. During assessment, additional information was gathered and some questions were modified. The participants were interviewed in their local language by trained data collectors (elementary school teachers) selected from the local area. Each interview was conducted during a house visit. Information concerning the socio-demographic characteristics of the participants was included in the questionnaires.

After completing the quantitative data collection, two FGDs (one with men and the other with women) comprising 8-10 men or women who were not involved in the individual interviews were conducted in the Hanekisna-Arado Kebele, Dubti District. Two comparable FGDs were conducted in the Angellele Kebele, Amibara District. These kebeles were randomly chosen from the kebeles included in the study. The discussions were carried out separately with men and women at different times on the same day. Specific topics concerning TBL were prepared as guidelines for discussion, moderated by the principal investigator and a trained health worker. The topics were presented in isolation, allowing adequate discussion on each topic. Responses were recorded using a notebook, translated into Amharic and then into English. Socio-demographic characteristics of the participants were recorded during the discussions.

### Data analysis

Collected data were translated into English, coded and double-entered into a data entry file using EpiData software V.3.1. The data were analysed using Stata version 8 (StataCorp LP). A Pearson chi-square test was used to evaluate the statistical significance of bivariate association of gender and selected covariates in each district. Univariable and multivariable logistic regression analyses were performed at a 5% significance level to investigate independent variables that were predictors of overall knowledge of TBL. The multivariable logistic regression model was developed by fitting all independent variables into the model. The effect of each of the variables (district, gender, age, education, occupation and marital status) on the overall knowledge of TBL was assessed by adjusting each variable for each of the other variables. The effect of cluster sampling was taken into consideration during the analysis. Differences were considered significant when p < 0.05.

Overall knowledge concerning TBL among the study participants was assessed using the following five points: (1) having information about TBL (direct or indirect); (2) ability to mention bacteria/germs as a cause of TBL; (3) mentioning that swelling(s) on the neck, followed by a lesion and scar, is a common symptom of cervical TBL; (4) knowing that TBL can be treated; (5) knowing that TBL is not normally transmitted from person to person. Responses to these questions were added together to generate a knowledge score ranging from 0 to 5. A score of one was given for correct responses; a score of zero was used for incorrect and 'do not know' responses. Using the mean score of the composite variable (mean = 2.8), the responses were categorised into high (score above mean value) and low (score below mean value) overall knowledge of TBL.

Information collected during the FGDs was translated into Amharic and entered into separate tables for women and men, according to the study area. Responses that reflected the common views of the discussants were selected and translated into English. The accuracy of the translation was checked by re-translating the data into Amharic and then the local language (Afargna) by another person. Responses were analysed for content analysis [17]. Briefly, similar responses given by the discussants in response to each topic of discussion were highlighted and sorted into one category for each topics. Ideas from each discussant were compared for similarities/differences and statements/conclusions were drawn for each of the topics discussed.

## Results

### Socio-demographic characteristics

A total of 818 participants [357 females (43.6%) and 461 males (56.4%), age range 18-70 years, mean age 36.9 years] were enrolled in the study from two areas. Three hundred and ninety-four (48.2%) participants were from the Dubti District and 424 (51.8%) were from the Amibara District. The majority of participants were pastoralists (71.6%), and the majority were illiterate (92.1%) (Table 1). Eighteen (10 men and 8 women, age range 24-70 years, mean age 40.7 years) participated in the FGDs held at the Hanekisna-Arado kebele, and 20 (10 men and 10 men, age range from 20-80 years, mean 43.9 years) participated in those held at the Angellele kebele. Among the 18 participants from Hanekisna-Arado, 13 (72.2%), 5 (27.8%) and 18 (100%) were pastoralists, agro-pastoralists and illiterate, respectively; of the 20 participants from Angellele, the corresponding figures were 15 (75%), 5 (25%) and 18 (90%).

**Table 1:** Socio-demographic characteristics of the study participants, Dubti and Amibara districts, Afar Region, north east Ethiopia, May 2009

Characteristic	Dubti; Number (%)	Amibara; Number (%)	Total (%)
Gender:			
Female	162 (41.1)	195 (46.0)	357 (43.6)
Male	232 (58.9)	229 (54.0)	461 (56.4)
Age (years):			
18-29	68 (17.3)	140 (33.0)	208 (25.4)
30-44	227 (57.6)	190 (44.8)	417 (51.0)
45-59	87 (22.1)	65 (15.3)	152 (18.6)
60+	12 (3.0)	29(6.8)	41 (5.0)
Martial status:			
Married	375 (95.7)	392 (92.5)	767 (94.0)
Other	17(4.3)	32 (7.5)	49 (6.0)
Ethnicity:			
Afar	394 (100)	423 (99.8)	817 (99.9)
Other	0 (0)	1(0.2)	1 (0.1)
Region:			
Muslim	394 (100)	424 (100)	818 (100)
Occupation:			
Pastoralist	260 (66.0)	326 (76.9)	586 (71.6)
Agro-pastoralist	134 (34.0)	98 (23.1)	232 (28.4)
Educational status:			
Illiterate	361(91.6)	392 (92.5)	753 (92.1)
Primary	19 (4.8)	11(2.6)	30 (3.7)
Secondary	4 (1.0)	2 (0.5)	6 (0.7)
Other (read & write)	9 (2.3)	20 (4.7)	29 (3.5)

### Knowledge of the cause, symptoms and treatment of TBL

Of the 818 participants, 742 (90.7%) reported that they had heard of or seen TBL (locally known as "Hulahule"). Of these 742 respondents, 739 (99.6%) mentioned that swelling(s) on the neck followed by a lesion and scar is a common symptom of the disease. However, only 11 (5 females and 6 males) individuals mentioned bacteria/germs as the causative agent. Other suggestions concerning the cause of TBL included food problems, cold air, chewing chat/smoking, dust, sunlight and dirty blood (Table 2). Three individuals suggested drinking raw milk was the cause of TBL. All discussants from both study areas mentioned swelling of the neck as the main symptom. Regarding the cause of the disease, hot air, strong sunlight and contamination of blood were the most frequently mentioned factors, and some discussants believed that the cause is similar to PTB (factors such as shortage of food, cold air or dust). A male discussant from Amibara stated that "a cow *infested by ticks usually gets a disease, and a person who consumed milk from that cow could develop TBL*."

**Table 2:** Communities' knowledge about cause, symptoms and treatment of TBL, Dubti and Amibara districts, Afar Region, north east Ethiopia, May 2009

Variable	Dubti			Amibara			
	**Female (%)**	**Male (%)**	**Total (%)**	**Female (%)**	**Male (%)**	**Total (%)**	**Total (%)**
Have you ever heard TBL?							
Yes	137(85.1)*	216(93.1)*	353(89.8)	173(88.7)*	216(94.3)	389(91.7)	742(90.7)
No	24(14.9)	16(6.9)	40(10.2)	22(11.3)	13(5.7)	35(8.3)	76(9.3)
Cause of TBL:							
Bacteria/germ	1(0.7)	2(0.9)	3(0.9)	4(2.3)	4(1.9)	8(2.1)	11(1.5)
Cold air	56(40.9)	80(37.0)	136(38.5)*	43(25.0)	47(22.3)	90(23.5)*	226(30.7)
Food problem	73(53.3)	107(49.5)	180(51.0)*	39(22.7)	41(19.4)	80(20.9)*	260(35.3)
Smoking, chewing	19(13.9)	30(13.9)	49(13.9)	41(23.8)	34(16.1)	75(19.6)	124(16.9)
Sun light	18(13.1)	34(15.7)	52(14.7)	26(15.1)	19(9.0)	45(11.7)	97(13.2)
Dust	12(8.8)	21(9.7)	33(9.3)*	46(26.7)*	37(17.5)*	83(21.7)*	116(15.8)
Hot air	5(3.6)	8(3.7)	13(3.7)	26(15.1)	22(10.4)	48(12.5)	61(8.3)
Dirty blood, milk, etc	7(5.1)	27(12.5)	34(9.6)	14(8.1)	17(8.1)	31(8.1)	68(9.3)
Do not know	36(26.5)	58(27.2)	94(26.9)	85(49.7)	100(47.4)	185(48.4)	279(37.9)
TBL is treatable:							
Yes	135(98.5)	211(97.7)	346(98.0)*	161(93.6)	206(95.4)	367(94.6)*	713(96.2)
No	0(0)	1(0.5)	1(0.3)	3(1.7)	2(0.9)	5(1.3)	6(0.8)
Do not know	2(1.5)	4(1.9)	6(1.7)	8(4.7)	8(3.7)	16(4.1)	22(3.0)
Treatment:							
Traditional medicine	30(22.2)	33(15.6)	63(18.2)*	82(50.9)	96(46.6)	178(48.5)	244(34.2)
Modern drug	98(72.6)	168(79.6)	266(76.9)*	77(47.8)	102(49.5)	179(48.8)	445(62.4)
Both	7(5.2)	10(4.7)	17(4.9)	1(0.6)	7(3.4)	8(2.2)	25(3.5)
Do not know	0(0)	0(0)	0(0)	1(0.6)	1(0.5)	2(0.5)	2(0.3)

*there was statistically significant difference

The majority (96.2%) of individual participants from both areas mentioned that TBL is a disease that can be treated with modern drugs (62.4%) or traditional medicine (34.2%). A higher proportion of participants from the Dubti area mentioned modern drugs as an effective treatment for TBL than participants from Amibara (76.9% vs. 48.8%, p < 0.001). Herbal medicine was suggested as the effective treatment by those participants who suggested traditional medicine (Table 2). In addition, most discussants were worried that there is no modern drug for the effective treatment of the disease, particularly in Ethiopia. However, discussants from both areas suggested that traditional medicinal plants and modern drugs could be used to treat the disease (if a patient goes to abroad, for example to Djibouti); although male discussants from Dubti concluded that traditional medicinal plants are the most effective means to treat TBL.

### Knowledge of the mode of transmission and prevention of TBL

The majority of individual respondents (79.7%) from both study areas believed that TBL can be transmitted from a patient to another person through bodily contact (67.2%) with a lesion or fluid from a lesion. A higher proportion of participants from Amibara than Dubti suggested that bodily contact was the main route of transmission of the disease (78.1% vs. 56.3%, p < 0.001). A larger proportion of participants from Dubti believed that coughing/breathing was a mode of transmission of the disease than participants from Amibara (63.1% vs. 40.7%, p < 0.001). In addition, some participants mentioned that the disease can be transmitted through sexual intercourse or by flies (Table 3). The participants believed that avoiding bodily contact, the exchange of clothes, sharing feeding materials, coughing/breathing and sexual intercourse can prevent transmission of the disease from a patient to another person. Bodily contact or exchanging clothes with a patient were suggested as the main modes of transmission by discussants from both study areas.

**Table 3:** Communities' knowledge about mode of transmission of TBL, Dubti and Amibara districts, Afar Region, north east Ethiopia, May 2009

Variable	Dubti			Amibara			
	**Female (%)**	**Male (%)**	**Total (%)**	**Female (%)**	**Male (%)**	**Total (%)**	**Total (%)**
TBL can be transmitted:							
Yes	112 (81.8)	183 (85.1)	295 (83.8)*	136 (79.1)	159 (73.6)	295 (76.0)*	590 (79.7)
No	17(12.4)	30 (14.0)	47 (13.4)	8 (4.7)	21(9.7)	29 (7.5)	76 (10.3)
Do not know	8 (5.8)	2 (0.9)	10 (2.8)	28 (16.3)	36 (16.7)	64 (16.5)	74 (10.0)
TBL transmitted through:							
Contact	69 (61.6)	97 (53.0)	166 (56.3)*	105 (76.6)	127 (79.4)	232 (78.1)*	398 (67.2)
Cough/breathing	67(59.8)	119 (65.0)	186 (63.1)*	57 (41.6)	64 (40.0)	121 (40.7)*	307 (51.9)
Sharing cups/feeding	31(27.9)	47 (25.7)	78 (26.5)	44 (32.1)	47 (29.4)	91 (30.6)	169 (28.5)
Other (sex, fly, cloths)	4 (3.6)	7 (3.8)	11(3.7)	5 (3.6)	15 (9.4)	20 (6.7)	31(5.2)
Do not know	3 (2.7)	6 (3.3)	9 (3.1)	4 (2.9)	3 (1.9)	7 (2.4)	16 (2.7)

*there was statistically significant difference

### Perception of communities concerning the public health importance of TBL

Table 4 presents the individual participants' perception concerning the public health importance of TBL in the study areas. The majority of respondents (71.6%) thought that TBL is rare in both study areas compared with PTB. However, a higher proportion of participants from Dubti than from Amibaba reported that the disease was a public health problem (32.6% vs. 17.0%, p< 0.000). In addition, a higher proportion of participants from the Dubti District (37.3% vs. 3.3%, p < 0.001) reported that the incidence of the disease has been increasing recently owing to a shortage of food (87.5%). Most participants from both areas (71.7%) said that TBL can affect male and females, and all age groups. However, a larger proportion of participants from the Dubti area than those from Amibara (91.1% vs. 62.6%, p < 0.001) reported that the disease mostly affected children under 15 years of age. In contrast, participants from Amibara claimed that TBL predominantly affects adults or old people (82.2% vs. 70.6%, p < 0.001). When participants were asked why they thought children were predominantly affected by the disease, many mentioned a lack of personal hygiene and a shortage of food as reasons, although the majority (78.9%) had no idea why this should be the case. Female participants from the Dubti area strongly argued that children less than 15 years of age are affected by TBL because of a lack of personal hygiene. The majority of respondents from Amibara (92.9%) had no idea why adults or old persons are predominantly affected by the disease. However, several suggested that old persons have poor resistance against disease, and that adults are normally involved in activities that expose them cold air, sunlight and dust, factors that they had previously suggested were associated with the disease.

**Table 4:** Communities' perception about the public health importance of TBL, Dubti and Amibara districts, Afar Region, north east Ethiopia, May 2009

Variable	Dubti			Amibara			
	**Female (%)**	**Male (%)**	**Total (%)**	**Female (%)**	**male (%)**	**Total (%)**	**Total (%)**
TBL is a health problem:							
Yes	49 (35.8)	66 (30.6)	115 (32.6)*	28 (16.2)	38 (17.6)	66 (17.0)*	181 (24.4)
Rare	84 (61.3)	146 (67.6)	230 (65.2)*	133 (76.9)	168 (77.8)	301 (77.4)*	531 (71.6)
No	4 (2.9)	4 (1.9)	8 (2.3)	12 (6.9)	10 (4.6)	22 (5.7)	30 (4.0)
Do not know	0 (0)	0 (0)	0 (0)	0 (0)	0 (0)	0 (0)	0 (0)
TBL is a public health problem since when:							
Since many years ago	85 (64.4)	129 (61.1)	214 (62.4)*	156 (96.9)	196 (95.1)	352 (95.9)*	566 (79.7)
Since recent years	46 (34.8)	82 (38.9)	128 (37.3)*	3 (1.9)	9 (4.4)	12 (3.3)*	140 (19.7)
Do not know	1 (0.8)	0 (0)	1 (0.3)	2 (1.2)	1 (0.5)	3 (0.8)	4 (0.6)
TBL mostly attacks:							
Male	10 (7.4)	31(14.5)	41(11.7)	12 (7.0)	29 (13.4)	41(10.6)	82 (11.1)
Female	7 (5.1)	6 (2.8)	13 (3.7)	8 (4.7)	7 (3.2)	15 (3.9)	28 (3.8)
Both	100 (73.5)	160 (74.8)	260 (74.3)	124 (72.1)	145 (67.1)	269 (69.3)	529 (71.7)
Do not know	19 (14.0)	17 (7.9)	36 (10.3)	28 (16.3)	35 (16.2)	63 (16.2)	99 (13.4)
TBL mostly attacks:							
Under 5 years	96 (70.1)	147 (69.0)	243 (69.4)*	88 (51.5)	130 (59.9)	218 (56.2)*	461 (62.5)
Children b/n 5 - 15 years	119 (86.9)*	200 (93.9)*	319 (91.1)*	104 (60.8)	139 (64.1)	243 (62.6)*	562 (76.2)
Adult under 60 years	103 (75.2)	144 (67.6)	247 (70.6)*	146 (85.4)	173 (79.7)	319 (82.2)*	566 (76.7)
Old person over 60 years	97 (70.8)	146 (68.5)	243 (69.4)*	141 (82.5)	170 (78.3)	311 (80.2)*	554 (75.1)
Do not know	6 (4.4)	2 (0.9)	8 (2.3)	15 (8.8)	16 (7.4)	31 (8.0)	39 (5.3)

*there was statistically significant difference

High overall knowledge of TBL was significantly associated with male study participants (adjusted OR, 1.82; 95% CI, 1.32 to 2.51, p < 0.001). Individuals aged between 45 and 59 years were 2.19 times more likely to have overall knowledge of TBL than other participants (adjusted OR, 2.19; 95% CI, 1.12 to 4.28, p = 0.022) (Table 5).

**Table 5:** Association of respondents' socio-demographic characteristics with respondents' overall knowledge of TBL, Dubti and Amibara districts, Afar Region, north east Ethiopia, May 2009

Characteristic	Overall knowledge of TBL			
	High, No(%)	Low, No(%)	Crude OR(95%, CI)	Adjusted OR(95%, CI)
District:				
Dubti	343 (88.4)	45 (11.6)	Reference	Reference
Amibara	361 (87.2)	53 (12.8)	0. 89 (0.31-2.59)	0.98(0.35- 2.74)
Gender:				
Female	294 (83.8)	57 (16.2)	Reference	Reference
Male	410 (90.9)	41 (9.1)	1.94(1.45- 2.56)	1.82 (1.32-2.51)
Age (years):				
18-29	174 (84.5)	32 (15.5)	Reference	Reference
30-44	357 (87.5)	51 (12.5)	1.29 (0.65- 2.57)	1.36 (0.72- 2.57)
45-59	138 (92.0)	12 (8.0)	2.12 (1.05- 4.25)	2.19 (1.12 - 4.28)
60+	35 (92.1)	3 (7.9)	2.15 (0.49- 9.44)	2.13 (0.55- 8.35)
Occupation:				
Pastoralist	505 (87.7)	71 (12.3)	Reference	Reference
Agro-pastoralist	199(88.1)	27 (12.0)	1.04 (0.45-2.37)	1.01 (0.49 - 2.15)
Educational status:				
Illiterate	646 (87.4)	93 (12.6)	Reference	Reference
Literate	58 (92.1)	5 (7.9)	1.67 (0.81-3.44)	1.64 (0.87 -3.12)
Marital status:				
Married	663 (88.2)	89 (11.8)	Reference	Reference
Other	40 (83.3)	8 (16.7)	0.67 (0.22 -2.07)	0.64 (0.21 - 1.93)

## Discussion

Tuberculosis (TB), pulmonary and extra-pulmonary (EPTB), is a major public health problem in developing countries [1]. However, there is little or no information concerning community awareness of EPTB. In this study, community knowledge and perception about the cause, mode of transmission, treatment, preventive methods and public health importance of TBL in pastoral communities in the middle and lower Awash valleys of Afar region was assessed. The results indicated a lack of information concerning the correct cause and mode of transmission of TBL and preventive methods. Multiple factors including shortage of food, cold air, smoking/chewing, hot air, strong sunlight and contamination of blood were suggested as causes of the disease by interviewees and discussants. This diversity of perceptions concerning the cause of TBL in the present study is comparable to those reported in previous studies carried out in Ethiopia [13-16,18,19] and other countries including Vietnam [20], Tanzania [17] and Kenya [21]. The community perception concerning the role of starvation and smoking/chewing as the cause of TBL could have implications as they will be thought to facilitate the development or the spread of TB [22-24]. In addition, misconceptions concerning the correct causative agent and modes of transmission could affect patient attitudes towards health-seeking behaviour, and contribute to the spread of the disease in pastoral communities of the study areas.

However, the results of this study indicated that the majority of the interviewees (90.7%) and all the discussants were familiar with the disease as they explained TBL (locally known as "Hulahule") by its symptoms. This high level of community awareness of the symptoms of the disease is important; recognizing swelling on the neck, axillary or inguinal in the early stage will encourage individuals to consult a health service.

Studies have suggested that Mb infection in humans is the main cause of the various forms of EPTB, while consumption of raw milk is the predominant potential source of infection [5,25-27]. However, the present pastoral communities had different perceptions regarding the transmission of TBL, believing that the disease can be transmitted from a patient to another person through bodily contact with the lesion of a patient or through coughing/breathing. This lack of awareness about the role of milk in the epidemiology of the disease could be problematic for pastoralists as raw milk is the daily food in these regions and they have close contact with animals, which could increase the risk of contracting Mb infection. Thoen et al. [27] underlined that human TB, due to infection with Mb, continues to be a public health problem predominantly in infants and children from countries where bovine TB is common. This review suggested that transmission of the disease could be reduced by boiling milk before ingestion and regular pasteurisation of dairy products. The strategy of routinely testing animals for Mb infection and slaughtering infected animals is not feasible in the present study areas. However, public health education focussing on raising the Afar pastoral community's awareness of human TB due to infection with Mb, possible sources of infection, modes of transmission and preventive methods, particularly boiling milk before ingestion, is of great importance.

Infection with Mtb can result in TBL, and patients with TBL can have positive sputum smears or culture results despite normal radiological chest findings [6,28-30]. Therefore, patients with EPTB could transmit TB to others [31], reflecting the knowledge of pastoralists in the present study areas. In the case of TBL, rupturing of cervical swollen lymph nodes produces a discharge that may contain the bacteria, and this can be contagious to other individuals, although it may not directly cause TBL. The concern of study participants that TBL could be transmitted through various routes should not be neglected, and further studies are required to elucidate the sources of infection, the modes of transmission and the mycobacterial species that cause EPTB in Afar pastoralists.

The WHO recommended that in countries with a high burden of TB, a single dose of bacillus Calmette-Guérin (BCG) vaccine should be given to healthy infants as soon as possible after birth to reduce the development of the primary form of TB [32]. In Ethiopia, the national BCG vaccination coverage at birth is estimated to be between 74 and 83% [33,34]. However, according to the recent national expanded program on immunization (EPI) coverage survey report in Ethiopia, BCG vaccination coverage in the Afar Region was approximately 9.3% [34]. This low coverage could contribute to the high prevalence of TB in the region and to the development of the primary form of the disease in children. This suggests that expansion of the BCG vaccine coverage program in the Afar Region could be important when designing TB control strategies.

The expansion of EPTB has been associated with the HIV/AIDS pandemic [2,35]. However, the majority of study participants claimed that TBL is one of the ancient diseases in Afar and that its epidemiology has remained unchanged. This perception implies an association between the disease and the lifestyle of pastoralists, confirming an earlier suggestion by Mfinanga et al. [8]. The present findings revealed that male study participants have a relatively greater biomedical knowledge of TBL than females, supporting the findings of other studies concerning PTB [16,15]. The low knowledge that women have concerning this disease could affect their health-seeking behaviour. Therefore, health education aimed at raising the awareness of women about TBL is crucial in the present study areas.

Among traditional medicines, herbal medicines are considered the most popular for the treatment of TB in several African countries including Ethiopia [18,36,37]. In the present study, a considerable number of the interviewees and almost all the discussants thought that traditional remedies, particularly herbal medicines, are more effective for treating TBL than modern drugs; this is a different view from the effective treatment suggested for PTB by the same study participants [16]. Men discussants from the Dubti area concluded that applying a medicinal plant to the affected area of the body can cure the disease within a short period of time. The communities' experience of and preference for herbal medicines to treat TBL could be related to the pastoral way of life or reflect the true effectiveness of herbal remedies. Therefore, additional research is required to investigate the anti-mycobacterial properties of herbal medicines used by the Afar people to treat TBL.

In this study, the use of systematic random sampling to select the study participants using list of households rather than employing simple random sampling could be a limitation. However, as far as we are aware, there is no underlying trend related to outcome variables that could have affected the findings. In addition, this study is one of the few that provides specific information concerning the pastoral community's knowledge of TBL, and this could be useful when designing appropriate control strategies for the disease, particularly in the present study areas and in settings where TBL is common.

## Conclusions

The results of this study revealed that pastoral community members from the study areas had low biomedical knowledge of the cause, source of infection and the mode of transmission of TBL. Moreover, men had better overall knowledge of the disease than women. A large number of community members also believed that herbal medicines are the most effective treatment for TBL. Therefore, TB control programs in the Afar region must introduce current biomedical knowledge of the disease, while further studies are required to investigate the traditional medicinal plants used by the Afar pastoralists to treat it.

## Competing interests

The authors declare that they have no competing interests.

## Authors' contributions

ML designed the study, participated in data collection, analysis and drafted the manuscript. GA, participated in study design, data collection, analysis and write-up. GM participated in study design, data collection and write-up. GMD, participated in study design, data analysis and interpretation. GB involved in study design and critically revised the manuscript. FA involved in study design, data analysis and write-up of the manuscript and critically revised the manuscript. All authors read and approved the final manuscript. ML is the guarantor of the paper.

## Pre-publication history

The pre-publication history for this paper can be accessed here:

http://www.biomedcentral.com/1471-2458/11/157/prepub

## Supplementary Material

Additional file 1 **Questionnaires administered in the study**. The questionnaire has all the questions that were used to collect quantitative data reported within the manuscript. Click here for file
