# Supplementary material for: Knowledge of cervical tuberculosis lymphadenitis and its treatment in pastoral communities of the Afar region, Ethiopia
Source: BMC Public Health. 2011 Mar 9;11:157. doi: 10.1186/1471-2458-11-157 (PMC3062609; doi:10.1186/1471-2458-11-157)
Supplement: Additional file 1 — Questionnaires administered in the study. The questionnaire has all the questions that were used to collect quantitative data reported within the manuscript. [file 1471-2458-11-157-S1.DOC]

Questionnaire for assessment of awareness about Tuberculosis Lymphadenitis (TBL)) in rural pastoral communities of Afar Region

**Part I. Socio- demographic characteristics of the respondents**

1. Name/ code of the respondent_________________________________

District **____________________________**Kebele**__________________** Village**________________________________-** House No**._____________**

1. Sex **:** 1= Male 2= Female
2. Age (Year): ____________

4. Ethnicity**:** 1= Afar 2= other specify ________________________________

5. Religion 1= Muslim 2= other specify _________________________________

6. Marital status 1= Married 2= single 3 = divorced 4= widowed

7. Educational status: 1= illiterate 2= read & write 3 = only read 4= primary (1-8) 5= secondary (9-12) 6= other (specify)_______________________-

8. Occupation: 1= Nomadic pastoralist 2= Agro-pastoralist 3= Merchant

4= Daily laborer 5= other (specify) ____________________________

9. Duration of residence in the area/village _______________ (years)

### Part II. Questionnaire about Knowledge of TBL

1. Have you ever seen/heard a person who sick from TBL (Hule-Hule in Afar language)

1= Yes 2= No

2. If yes, what do you think about the cause of this disease? **(do not read the alternatives but circle it if the respondent will mention it)**

1= Bacteria/germ 2= cold 3= shortage of food 4= smoking, chewing, drinking

5= hot climate 6= sun light 7= drinking raw milk

8= any other____________________________________

3. What is/are the common symptom (s) of TBL?

**____________________________________________________________________________________________________________________________________________**

4. Does this disease transmit from a patient to other person?

1= yes 2= No 3= Do not know

5 . If yes, how it can be transmitted from a patient to other person ? **(do not read the alternatives but circle it if the respondent will mention it)**

1= through breathing, cough 2= contact with wound or pus 3= sharing drinking /feeding materials with patient 4= Sharing clothes with a patient 5= any other _____________________________

6. Do you think that the transmission of TBL is preventable?

1= yes 2= no 3= do not know

7. If yes , preventive methods? **(do not read the alternatives but circle it if the respondent will mention it)** 1= avoid sharing cups with a patient 2= Do not cough/ sneeze at other people 3= Do not share clothes with a patient 4= use separate room for patient 5= avoiding body contact with a patient 6= any other_____________________________

8. Does this disease have a treatment?

1= Yes 2= No 3= Do not know

9. if yes, effective treatment for TBL?

1= Traditional medicine 2 = Modern drug 3= both 4= do not know

10. If traditional medicine, what type of treatment is it?

1= Medicinal plants 2= Food (specify) __________________

3= any other _____________________

**Part III. Assessment of Perception of Communities about Public Health Importance of TBL**

1. . Is TBL a major health problem in this area when compared to PTB ?

1= Yes 2 = No 3= Rare 4=Don’t know

2. If yes, since when the disease is becoming a health problem in this area?

1= since recent years 2 = since many years 3= Do not know 4= any other_____________________________________________________________

3. If since recent years, what factors do you think to contribute to its expansion ? (**do not read the alternatives but circle it if the respondent will mention it)**

1= HIV/AIDS 2= Poverty 3= climate change 4= Increasing of habits like smoking, chewing , drinking 5= Habit of drinking raw milk

6= any other _____________________________________________

4. In this area, TBL mostly attacks

1= children under 5 years 2= children 5-15 years 3= adults 4= very old people (over 60 years) 5= all 6= do not know

7= any other ______________________________

5. If one of the above age groups (e.g children under 5 years or old people), why do you think that TBL mostly attacks this age group? ________________________________________________________________________

6. TBL mostly attacks :

1 = male 2= female 3= both 4= do not know 5= any other _______________________________

7. If male or female why ? _______________________________________________

8. If you have any other comments or suggestion

Thank you
